# Supplementary material for: Realization of two-dimensional discrete time crystals with anisotropic Heisenberg coupling
Source: Nat Commun. 2026 Jan 28;17:605. doi: 10.1038/s41467-025-67787-1 (PMC12852674; doi:10.1038/s41467-025-67787-1)
Supplement: Supplementary file 1 — Supplementary Information [file 41467_2025_67787_MOESM1_ESM.pdf]

Supplementary Information for
"Realization of Two-dimensional Discrete Time Crystals with Anisotropic Heisenberg Coupling"

Eric D. Switzer,[1,2,3,*] Niall F. Robertson,[4] Nathan Keenan,[5,4,6] Ángel Rodríguez-Alcaraz,[7,8] Andrea D'Urbano,[4] Bibek Pokharel,[9] Talat S. Rahman,[3,1] Oles Shtanko,[10,†] Sergiy Zhuk,[4] and Nicolás Lorente[1,7,‡]

[1]*Donostia International Physics Center (DIPC), 20018 Donostia-San Sebastián, Euskadi, Spain*
[2]*Nanoscale Device Characterization Division, National Institute of Standards and Technology, Gaithersburg, Maryland 20899, USA*
[3]*Department of Physics, University of Central Florida, Orlando, Florida 32816, USA*
[4]*IBM Quantum, IBM Research Europe - Dublin, IBM Technology Campus, Dublin 15, Ireland*
[5]*Department of Physics, Trinity College Dublin, Dublin 2, Ireland*
[6]*Trinity Quantum Alliance, Unit 16, Trinity Technology and Enterprise Centre, Pearse Street, D02 YN67, Dublin 2, Ireland*
[7]*Centro de Física de Materiales CFM/MPC (CSIC-UPV/EHU), 20018 Donostia-San Sebastián, Euskadi, Spain*
[8]*Universidad del País Vasco/Euskal Herriko Unibertsitatea UPV/EHU. Department of Polymers and Advanced Materials: Physics, Chemistry and Technology. Faculty of Chemistry, University of the Basque Country, 20018 Donostia-San Sebastián, Spain.*
[9]*IBM Quantum, IBM T.J. Watson Research Center, Yorktown Heights, NY 10598, USA*
[10]*IBM Quantum, IBM Research – Almaden, San Jose, California 35120, USA*

CONTENTS

I. Device details S1

II. Classical simulation S2
A. Matrix Product State simulations S6
B. Two-dimensional Tensor Network State simulations S8

III. Signal recovery from noisy observables S9
A. Spin ordering S9
B. Correlations S13
C. Hamming distance S15

IV. Trends in order parameters derived from raw data S16
A. Quantum Fisher information order parameter S16
B. Time correlation order parameter S17
C. Edwards-Anderson spin glass order parameter S17

V. Discrete vs Continuous Time S18

Supplementary References S20

I. DEVICE DETAILS

For our experiments, we used `ibm_fez`, a Heron r2 processor. Heron r2 processors comprise 156 fixed-frequency transmon qubits with tunable couplers on a heavy-hex lattice layout. At the time of usage, IBM Fez could implement 195,000 circuit layer operations per second. Of the 156 qubits on Fez, 144 qubits were chosen as our data qubits. Figures S1 and S2 highlight the data qubits such that the vertex of the connectivity graph shows the T_2 coherence times for the qubits and the edges show the error rates of two qubit gates. Figures S1 and S2 correspond to the

---

* eric.switzer@nist.gov
† oles.shtanko@ibm.com
‡ nicolas.lorente@ehu.eus

quantum backend's properties on the two data collection days - July 27th 2024 and December 3rd, 2024. The detailed device specification for all the data collection days are shown in Supplementary Table I. The qubit selection for the MPS labelings of 2×2, 3×3, 3×7 heavy hexagons are shown in Figure S6. Let us consider the device specifications for our data qubits for December 3rd 2024. The mean readout error was 2.3 % and the median was 1.7 %. The higher mean indicates an asymmetry with some high readout error qubits skewing the distribution. Likewise, the dephasing time T_2 had a mean of $90\,\mu$s and a median of $84\,\mu$s. In contrast, the relaxation times T_1 had a more symmetric distribution with both mean and median times of $130\,\mu$s. As expected, the dephasing times are lower than the relaxation times. The single qubit gates had mean error rates of 3.0×10^{-4} and median 2.6×10^{-4}. A similar qubit-wise asymmetry as discussed above was seen here as well. However, the difference between the mean and the median is not substantial. Lastly, the two-qubit gate error had a mean of 1.8×10^{-2} and median 2.9×10^{-3}.

An independent, observable-dependent quantification of noise can be obtained by evaluating the observable in the Clifford regime. At this point, the noiseless values of $|\Delta(t)|$ and $\chi(t)$ in Eqs. (5) and (6) in the main text both equal unity, which allows us to isolate the impact of noise. An example of this analysis is shown in Fig. S3 for $\Delta(t)$ at the Clifford point 2 in Fig. 3 in the main text ($\epsilon = 0$, $\phi = \pi/2$). The data reveals two distinct effects of noise: (i) a decay of the signal due to coherence loss, and (ii) a systematic offset arising from the non-unital component of the noise (amplitude damping). Fig. S3 indicates a decoherence time of approximately $T \approx 15$ Floquet cycles, corresponding to $D \approx 135$ two-qubit gate layers. The offset is approximately $\delta \approx -4 \times 10^{-2}$, which requires the dedicated error-mitigation strategy described in Section III.

Date	T_1 (μs)	T_2 (μs)	RO (10^{-2})	1Q (10^{-4})	2Q (10^{-3})
July 26th, 2024 (Mean)	110	90	2.4	3.6	18.0
July 26th, 2024 (Median)	100	94	1.6	2.9	3.0
July 27th, 2024 (Mean)	120	93	2.4	3.0	18.0
July 27th, 2024 (Median)	110	91	1.7	2.6	3.0
December 3rd, 2024 (Mean)	130	90	2.3	3.0	18.0
December 3rd, 2024 (Median)	130	84	1.7	2.6	2.9
December 11th, 2024 (Mean)	120	100	2.2	3.1	29.0
December 11th, 2024 (Median)	120	95	1.6	2.7	3.0
December 17th, 2024 (Mean)	140	100	2.1	2.9	18.0
December 17th, 2024 (Median)	130	97	1.7	2.4	2.9
December 18th, 2024 (Mean)	140	110	2.2	3.7	23.0
December 18th, 2024 (Median)	140	100	1.6	2.5	3.0

Supplementary Table I. Device specifications, in particular relaxation times T_1, dephasing time T_2, readout (RO), single-qubit (1Q) errors and two-qubit (2Q) errors rates are reported for the dates when the quantum backend was accessed for data collection.

Figure	Geometry	T_{max}	N_q	N_{shots}	N_S	N_{cs}	Selected Qubits	Start Date of Runs
Fig. 2	2x2	50	35	20000	1	4	Fig. S5	December 11th, 2024
Fig. 2	3x3	50	68	20000	1	6	Fig. S5	December 3rd, 2024
Fig. 2	3x7	50	144	20000	1	14	Fig. S2	December 3rd, 2024
Fig. 3	2x2	30	35	5000	33	165	Fig. S4	July 26th, 2024
Fig. 3	3x3	30	68	5000	33	165	Fig. S4	July 27th, 2024
Fig. 3	3x7	30	144	5000	33	165	Fig. S1	July 20th, 2024
Fig. 4	2x2	30	35	5000	33	165	Fig. 1	December 18th, 2024
Fig. 4	3x7	30	144	5000	33	165	Fig. 1	December 17th, 2024

Supplementary Table II. Details of each run used for data for a corresponding figure in the main text. T_{max} is the maximum number of Floquet cycles, N_q is the number of physical qubits, N_{shots} is the number of shots for each circuit, N_S is the total number of sessions for the run, N_{cs} is the total number of circuit splits used for the run, the selected qubits corresponds with the corresponding figure in this Supplementary, and the state date of the runs is for the first session of the run.

II. CLASSICAL SIMULATION

We used two classes of tensor network-based algorithms for our classical simulations. The first class utilizes Matrix Product States (MPS) along with established algorithms for updating, truncating, and contracting the MPS. The second class involves techniques based on two-dimensional tensor network states, which are designed to align with the

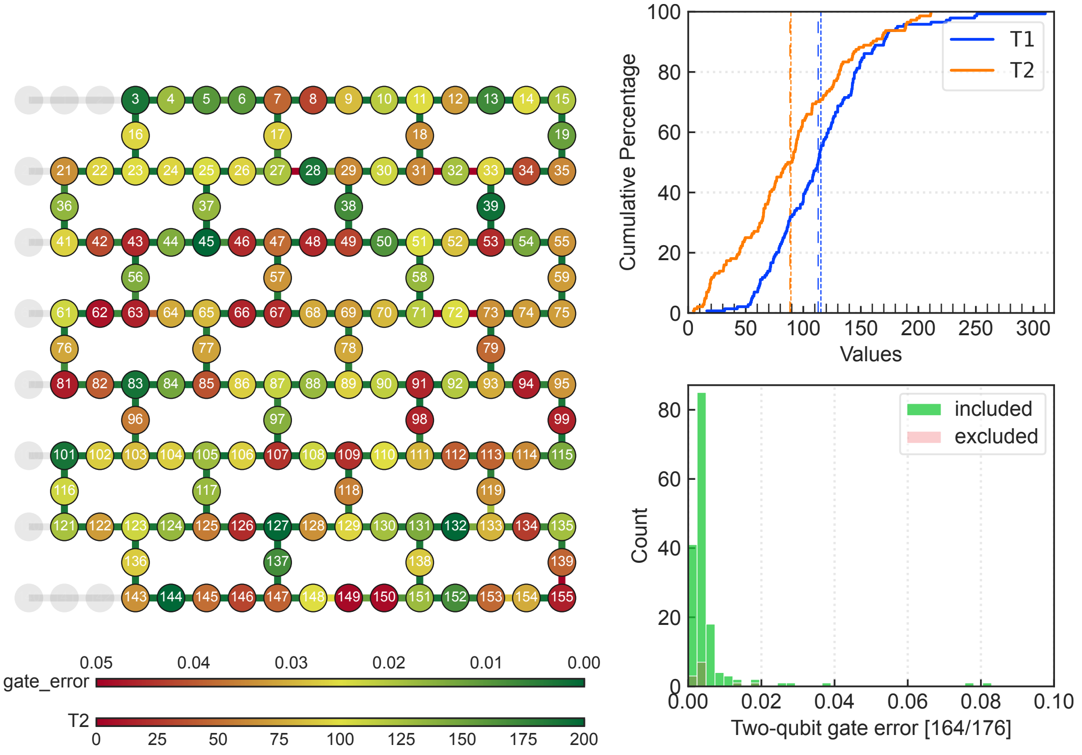

Figure S1. Device layout showing the data qubits for the 3x7 geometry and their specifications for our experiment on `ibm_fez` for July 27th, 2024 corresponding to the data shown in Fig. 3 in the main text. Left panel: Data qubits and the corresponding edges are highlighted according to their measured error rates (T_2 and two-qubit gate error). Top right panel: Coherence times T_1 and T_2 are shown using a cumulative distribution plot. Mean and median are highlighted using dotted and dashed lines. Bottom right panel: Two-qubit error rates are shown in a histogram; included and excluded qubits are plotted separately.

topology of the decorated hexagonal lattice. In this case, we adopt algorithms inspired by Belief Propagation[1,2] to update the two-dimensional tensor network. Further details on both classes of methods are provided in Sections II A and II B below.

The MPS representation provides one of the most common and well-understood techniques for classical simulation, primarily applied to one-dimensional systems. The success of these methods ultimately boils down to two key properties of MPS. First, MPS contraction is efficient; more precisely, the computational cost of contraction scales as χ^3,

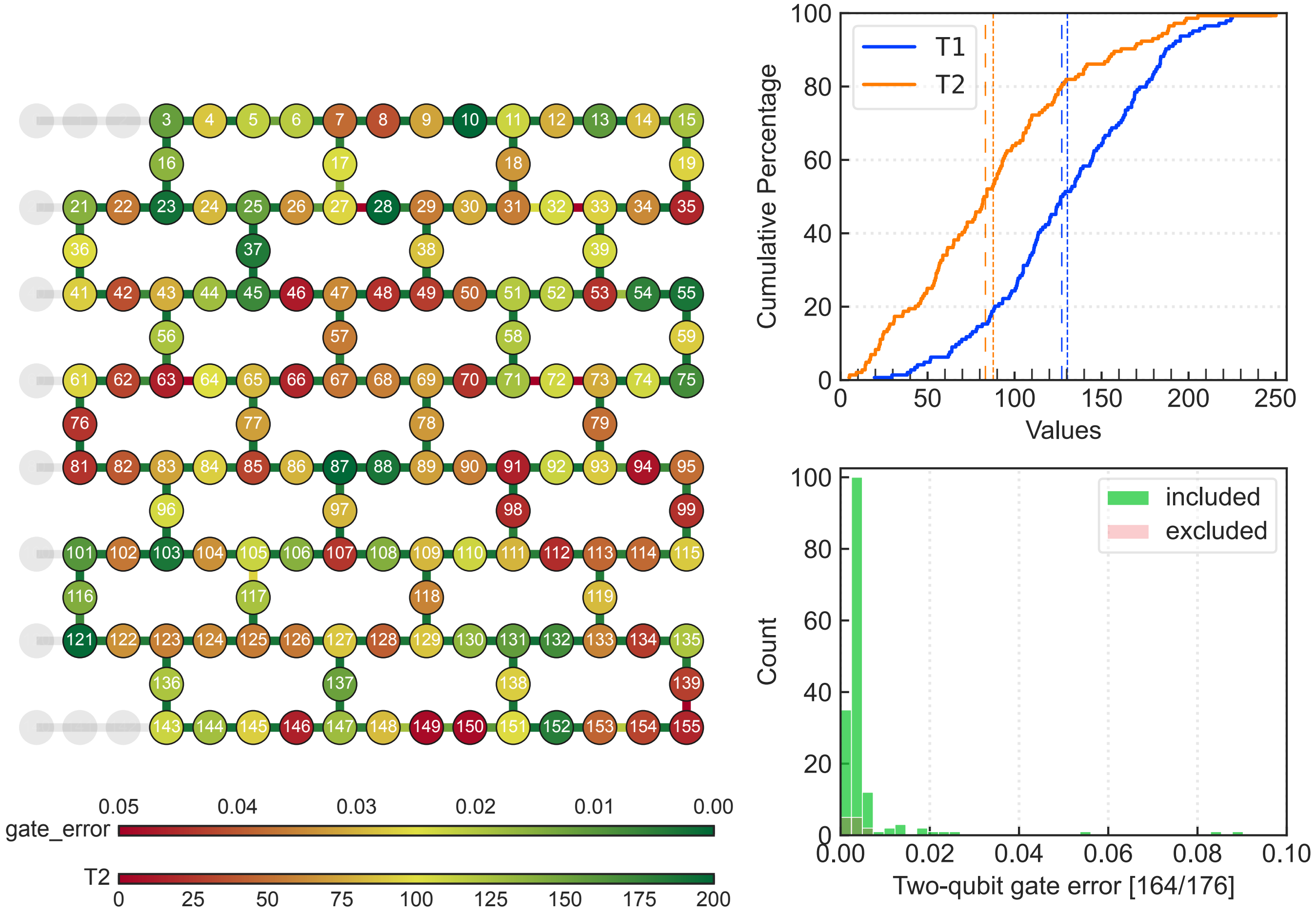

Figure S2. Device layout showing the data qubits for the 3x7 geometry and their specifications for our experiment on `ibm_fez` for December 3rd, 2024 corresponding to the data shown in Fig. 2 in the main text.

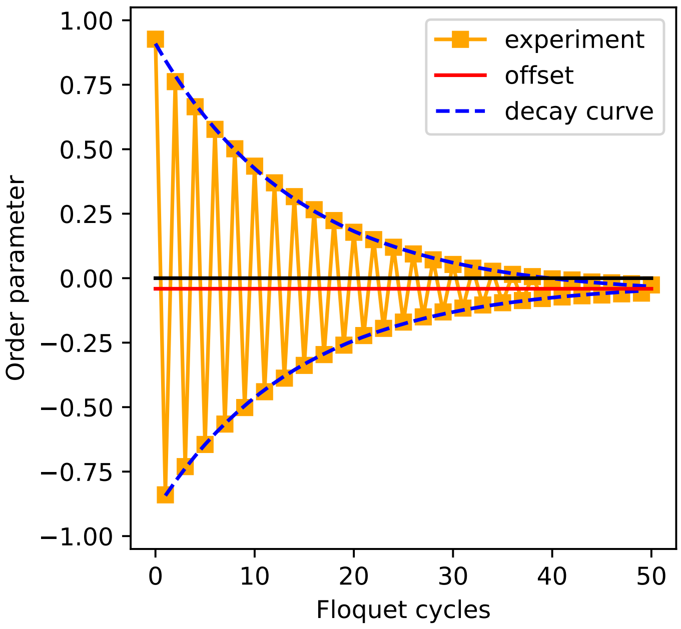

Figure S3. Quantitative noise analysis at the Clifford point (marked as Clifford 2 in Fig. 3 in the main text), used as a reference for error mitigation. In noiseless simulations, the order parameter alternates as $\Delta(t) = (-1)^t$, where t is the number of Floquet cycles. With noise, the observed signal takes the form $\Delta_{\text{noisy}}(t) = \Delta(t)f(t) + \delta$, where $f(t)$ accounts for decoherence noise (dashed blue curves) and δ is an offset due to amplitude damping noise (red line); see Eq. (S.5). The solid black line corresponds to zero signal. Approximating $f(t) \approx Ae^{-t/T}$, with T the characteristic decay time, allows extraction of the parameters $A = 0.973$, $T = 14.5$, and $\delta = -0.063$ (even cycles) and $\delta = -0.019$ (odd cycles), providing a quantitative characterization of the noise. These values are subsequently used to mitigate errors at non-Clifford points (see Section III).

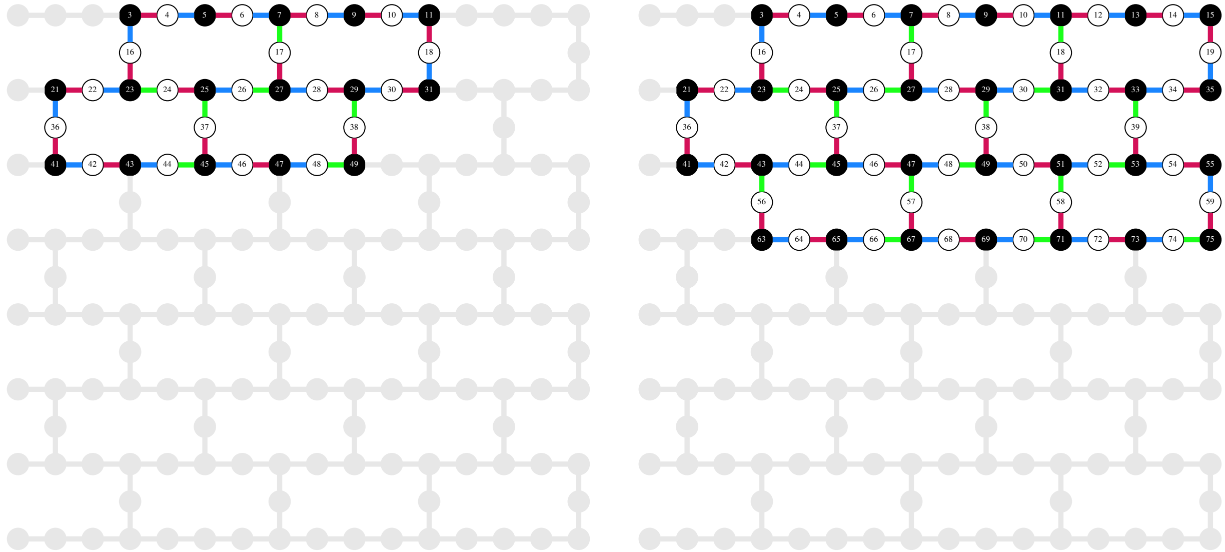

Figure S4. Selected device qubits for 2×2 and 3×3 geometries corresponding to Fig. 3 in the main text.

where χ is the maximum bond dimension of the network. Second, tensor network states generally possess a redundant

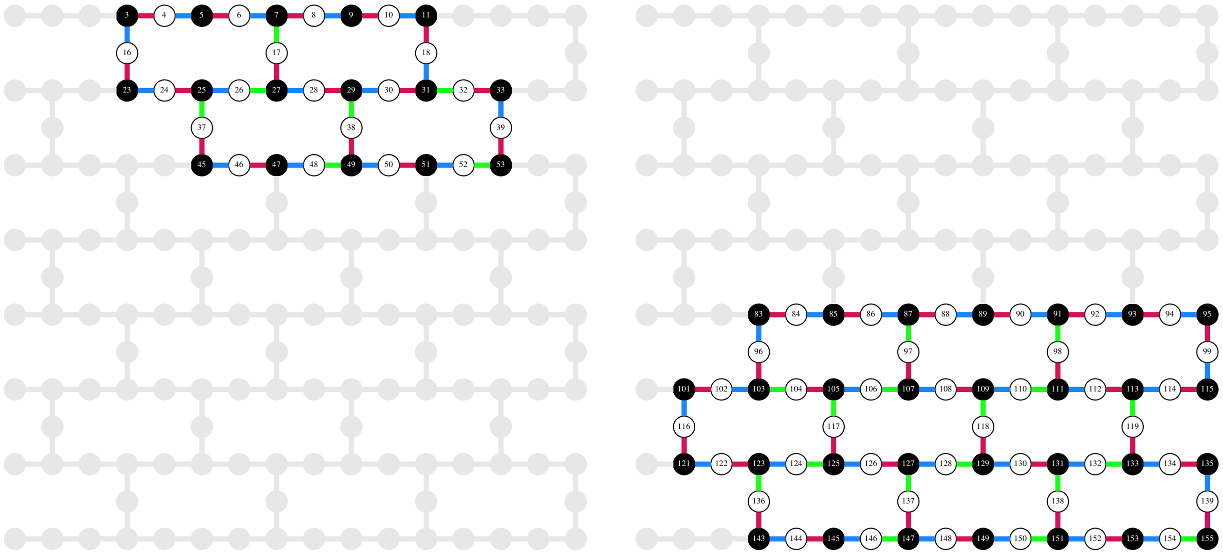

Figure S5. Selected device qubits for 2×2 and 3×3 geometries corresponding to Fig. 2 in the main text.

degree of freedom known as gauge freedom, meaning that for a given quantum state, the choice of tensors forming the network representation of the state is not unique. By applying a "gauge transformation," one can transform one tensor network into another with more favorable properties without affecting the underlying physical state. If the tensor network under consideration is an MPS, it is always possible to find a gauge transformation that converts the MPS into a "canonical form" such that each bond of the network defines an orthogonal basis. This allows for the truncation of the bond dimension at any bond in the network in an optimal way and with a controlled truncation error, see Ref.[3] for a review. The MPS representation can also be extended to models in two or higher dimensions by 'unrolling' them onto a single dimension. However, this approach introduces long-range interactions within the model, rendering standard algorithms, such as TEBD[4], inapplicable. To deal with these long range interactions, one can use approaches such as TDVP[5,6], the $W^{I,II}$ method[7], or the direct construction of the Matrix Product Operator (MPO) representing the time evolution operator, see Section II A below.

One can avoid the introduction of long-range interactions by using a two-dimensional tensor network state that matches the topology of the Hamiltonian under consideration. However, there are two primary obstacles facing two-dimensional tensor network states. First, the contractions required to calculate meaningful quantities, such as expectation values of observable quantities, scale exponentially with the size of the smallest spatial dimension[8]. Second, while methods exist to gauge-transform a general two-dimensional tensor network into a canonical form analogous to that of MPS[1,9,10], these higher-dimensional canonical forms do not allow for optimal or even well-controlled truncations of the bond dimension, see Section II B below.

In Figures S10 and S11 we compare the results for the single site polarizations and nearest neighbor two point correlators obtained by the two classical simulation methods, i.e., Matrix Product State simulations and two-dimensional tensor network state simulations with Belief Propagation (BP). In both figures, we compare the results for a number of different bond dimensions. Note that in all the plots there is excellent agreement between the methods at early to intermediate times. However, the results from the two methods begin to diverge from each other at later times. Furthermore, we observe that, at these later times, the MPS based simulations often struggle to converge with increasing bond dimension, whereas this is not the case for the two-dimensional tensor network state simulations which were updated and contracted with Belief Propagation.

A. Matrix Product State simulations

To apply MPS-based algorithms to two-dimensional models, we first 'unroll' the two-dimensional model onto one dimension, i.e. assign an integer value between 0 and $N - 1$ to each of the N sites in the two-dimensional model. This assignment is illustrated in Figure S6. The unrolling procedure comes at the expense of introducing long-range interactions. For example, the qubits with the MPS labels 1 and 22 in the top left panel of Figure S6 are nearest neighbors on the two-dimensional decorated hexagonal graph but are well separated on the MPS chain. A gate

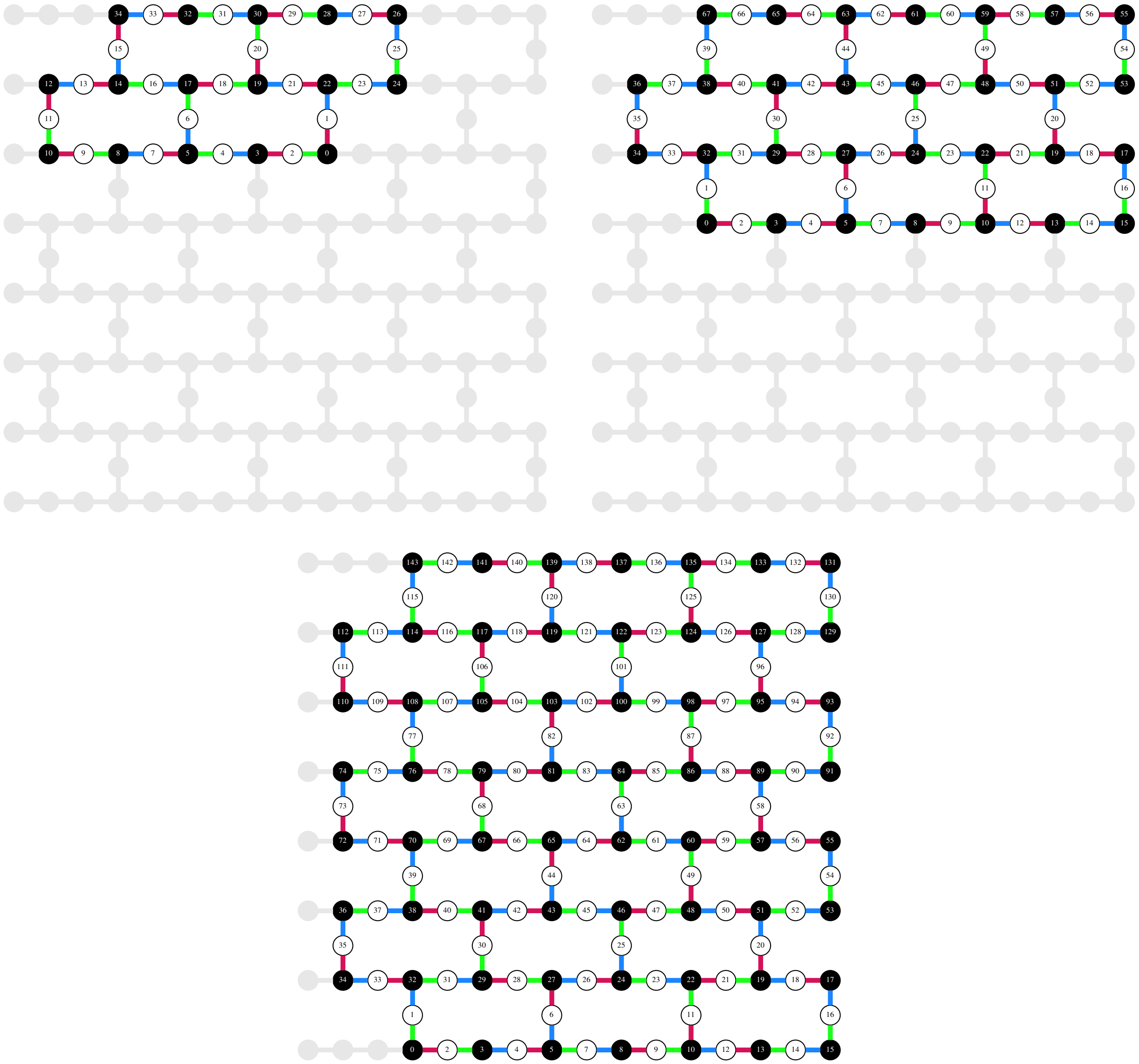

Figure S6. The MPS labeling of the system with 2×2, 3×3 and 3×7 heavy hexagons.

that acts on nearest neighbors in the heavy-hex model thus becomes a long-range gate when applied to the MPS representing the quantum state. There is no unique way to unroll the two-dimensional graph onto one dimension, and the precise method used will affect the amount of entanglement (and hence the required bond dimension for a given precision) in the description of the state. Here, we use a labeling scheme similar to the one previously used in Ref.[2].

We implement the long-range gates by constructing the Matrix Product Operator (MPO) for each layer $U_F^{(k)}$ in U_F, see Eq. (1) in the main text. We can represent each two-qubit operator U_{ij} in Eq. (4) in the main text as an MPO with bond dimension $\chi = 4$. The bond dimension of each $U_F^{(k)}$ is determined by the number of overlapping bonds after unrolling onto one dimension. For example, a maximum bond dimension of 64 is required to store the MPO of any of the individual layers $U_F^{(j)}$ in the system with 2×2 heavy hexagons in Figure S6. In all our simulations, the initial quantum state is a product state, which can be represented as a trivial MPS with bond dimension 1. The MPOs representing $U_F^{(1)}$, $U_F^{(2)}$, and $U_F^{(3)}$ are then individually contracted with the MPS representing the quantum state, such that we never need to store the MPO representing U_F itself. We perform the MPO-MPS contractions using a standard SVD-based algorithm[3], in which the MPS is truncated to its maximum allowed value χ_{max}. The MPS simulations with different values of χ_{max} are compared in Figures S10 and S11 for one particular point in the phase diagram.

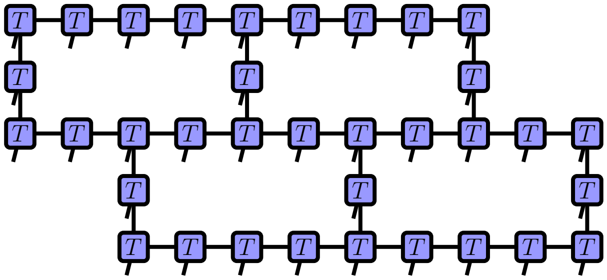

Figure S7. The two-dimensional tensor network state with 2×2 heavy hexagons.

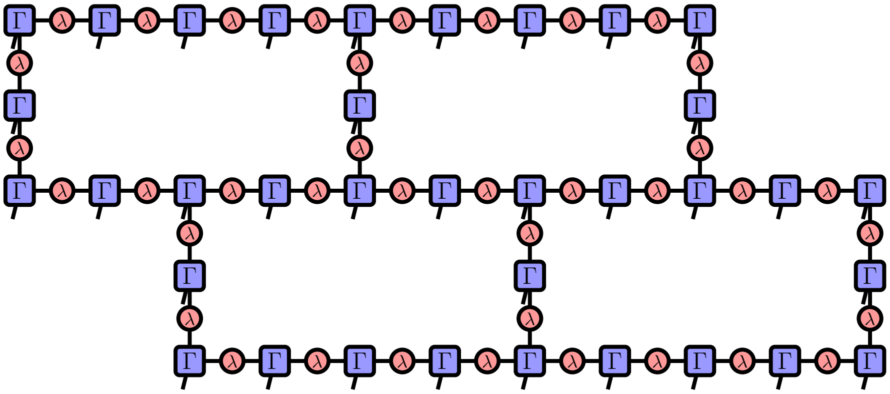

Figure S8. The quasi-canonical form of the the two-dimensional tensor network state with 2×2 heavy hexagons. Each site has a vertex tensor Γ and each edge has a bond tensor λ. We use a belief propagation algorithm to ensure that the Γ and λ tensors approximately satisfy the constraint shown in Figure S9 at each site.

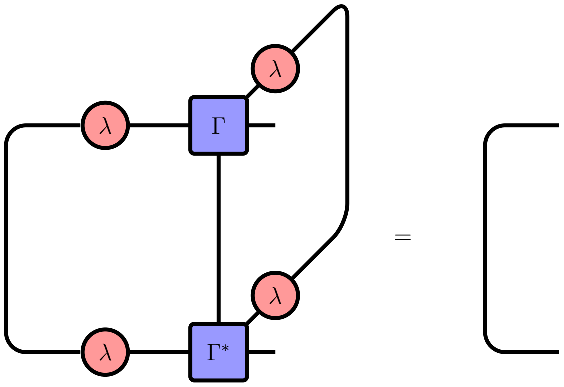

Figure S9. In the Vidal gauge, the vertex and bond tensors at each site satisfy the constraint defined by the contraction diagram shown above. This constraint can be stated as follows: at any given site, if one takes the vertex tensor Γ and contracts it with all but one of its bond tensors λ, the resulting tensor should be an isometry - i.e. contracting with its complex conjugate (LHS above) should result in the identity tensor (RHS above). The belief propagation algorithm applies a gauge transformation to the tensor network such that the equality shown in this contraction diagram is (approximately) satisfied at each site of the network. See[1] for more details.

B. Two-dimensional Tensor Network State simulations

We consider a two-dimensional tensor network state that matches the decorated hexagonal topology. In Figure S7, we show the structure of this tensor network for the case of 2×2 heavy hexagons. Each tensor has one physical leg

with two indices (corresponding to the $|0\rangle$ and $|1\rangle$ states of the qubit at each site) and either two or three virtual legs. Note that tensors on different sites are, in general, not equal to each other. The two-qubit operators U_{ij} in Eq. (4) in the main text can be implemented as gates that update pairs of nearest-neighbor tensors in the two-dimensional network. The application of a gate increases the bond dimension of the state, which we then truncate to the maximum bond dimension χ_{max}.

The error incurred by truncation depends heavily on the gauge of the tensor network, which we now discuss. For any given quantum state $|\psi\rangle$ described by a tensor network, the choice of tensors T in Figure S7 is not unique. For example, one can redefine each tensor T via the transformation $T \to X^{-1}TX$ for some matrix X. If the same X is used for each tensor T, then the state $|\psi\rangle$ described by the network remains unchanged under this gauge transformation, see Ref.[11] for a more extensive discussion of this point.

For MPS, it is well known how to construct the gauge for the network such that the error incurred by truncating the bond dimension is optimal, as defined by the fidelity between the state before and after truncation[3]. This is known as the canonical form or the "Vidal gauge." There is no simple way to construct a canonical form that can be truncated optimally for more general tensor network states, such as the one in Figure S7. Instead, one can construct a quasi-canonical form of a two-dimensional tensor network with properties similar to its one-dimensional counterpart, such that truncating the bond dimension produces errors low enough to be practical, even if they are not optimal. In this gauge, there are "vertex" tensors and "bond" tensors, referred to as Γ and λ, respectively, as shown in Figure S8. The vertex tensors have one physical leg and either two or three virtual legs, while the bond tensors have two virtual legs and can be represented as square matrices. The tensor network is said to be in the Vidal gauge if all tensors satisfy a particular constraint, i.e. that the combination of the vertex and bond tensors at each site forms an isometry, see Figure S9 for more details.

When truncating the bond dimension of the network, the truncation error is reduced when this truncation is performed while in the Vidal gauge. However, each application of a gate U_{ij} destroys the quasi-canonical form - the network thus has to be repeatedly "regauged" throughout the simulation. We use a belief propagation algorithm to put the tensor network back into its quasi-canonical form, i.e., so that the constraint in Figure S9 is (approximately) satisfied at each site. As discussed in Ref.[1,2], regauging after each application of a gate is superfluous and impractically expensive. We thus only regauge the network with belief propagation after each Trotter step.

Once we perform the updates to obtain a representation of the time-evolved quantum state, we need to extract meaningful quantities, such as the expectation values of observable operators. Doing so exactly would require the contraction of the full two-dimensional network—a notoriously difficult computational task[8]. However, we can calculate one-site observables by approximating the environment of the site as a separable product given by the λ matrices attached to the site in question. As discussed in detail in Ref.[1], when there are loops in the network—as is the case here—this approximation may not hold. Higher-weight observables can be calculated by applying a Clifford circuit U_c to the network such that the expectation value of a single-site observable, after the application of the Clifford circuit, is equal to the expectation value of the higher-weight observable in the original state[2]. More precisely, if we want to calculate the expectation value of, e.g., the two-point correlator Z_iZ_j, we find the Clifford circuit U_c such that $Z_iZ_j = U_c^\dagger Z_i U_c$ and hence $\langle\psi| Z_iZ_j |\psi\rangle = \langle\psi| U_c^\dagger Z_i U_c |\psi\rangle$. We use this method to obtain the BP results in Figure S11. This technique comes at the expense of increasing the number of gates applied to the network, thereby increasing the amount of entanglement in the state and the number of truncations that must be applied.

III. SIGNAL RECOVERY FROM NOISY OBSERVABLES

A. Spin ordering

We focus on the expectation values $s_i(t)$ of the Pauli-Z operators for spin i at discrete time t, along with the ideal spin ordering parameter $\Delta(t)$, defined as

$$s_i(t) := \langle Z_i(t)\rangle \equiv \langle\psi_t|Z_i|\psi_t\rangle, \qquad \Delta(t) = \frac{1}{N}\sum_{i=1}^{N} s_i(0)s_i(t), \tag{S.1}$$

where $|\psi_t\rangle$ represents the state vector of the noiseless evolution, and N denotes the number of qubits. In the experiment, however, we can only access the value $\tilde{s}_i(t)$, which incorporates the effects of noise, as well as initialization and measurement errors. As a result, the experimentally accessible order parameter reflects these noise contributions. Consequently, the measured order parameter takes the form

$$\Delta_{\text{noisy}}(t) := \frac{1}{N}\sum_{i=1}^{N} s_i(0)\tilde{s}_i(t), \tag{S.2}$$

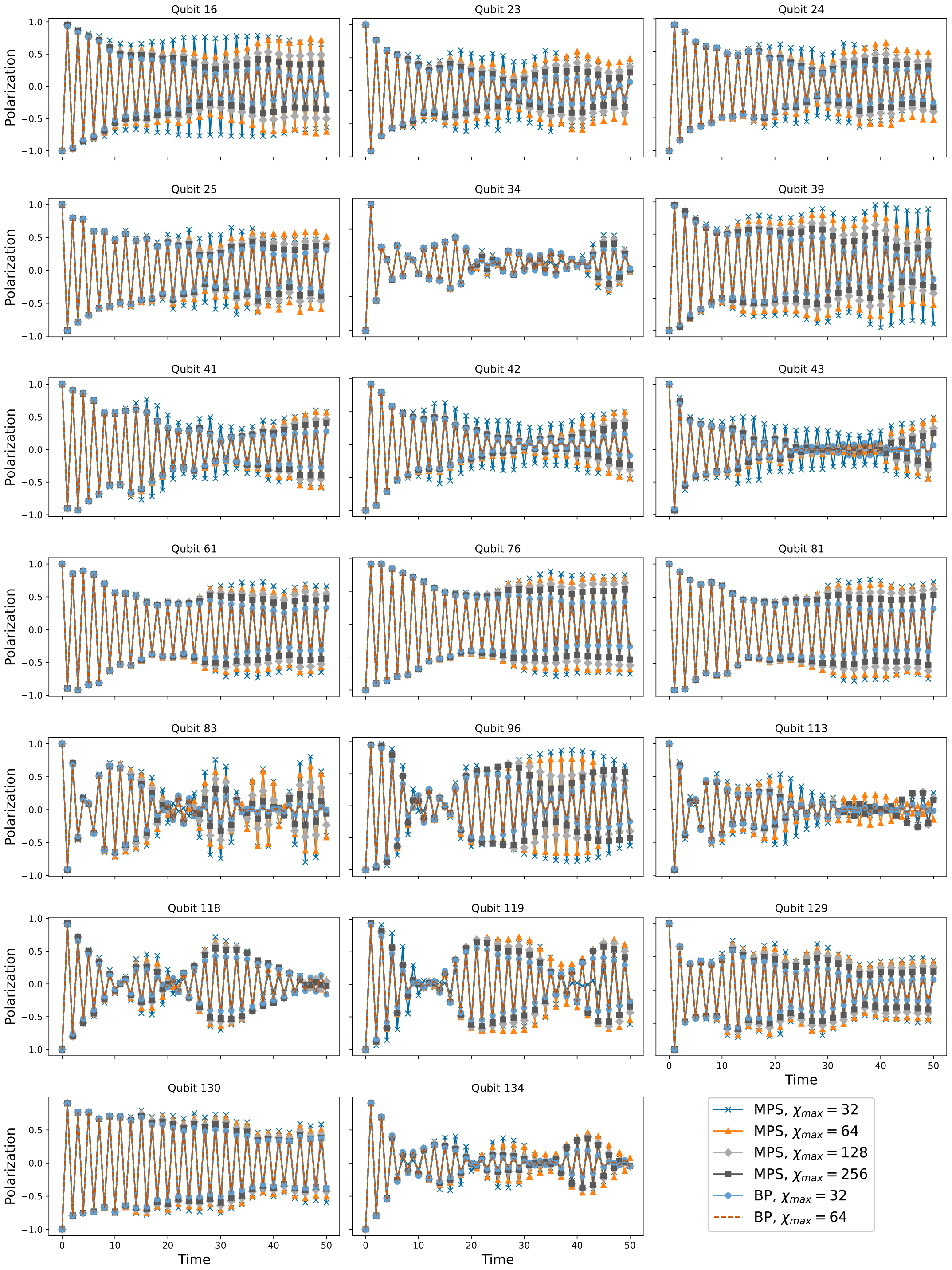

Figure S10. Classical simulation results for selected site polarizations in the 3x7 geometry for $\epsilon = 0.05$ and $\phi = 0.45\pi$. The simulations based on two-dimensional tensor network states with Belief Propagation (BP) converge with increasing bond dimension, whereas the MPS-based simulations don't always converge with the bond dimensions used here, particularly at later times.

where $s_i(0)$ corresponds to the initial spin projections.

While predicting individual $s_i(t)$ from their noisy counterparts $\tilde{s}_i(t)$ remains a challenging problem, several methods currently enable partial recovery of noiseless values, provided multiple copies of the noisy circuit and its modifications are available. These methods include twirling[12], zero-noise extrapolation[13,14], and probabilistic error amplification

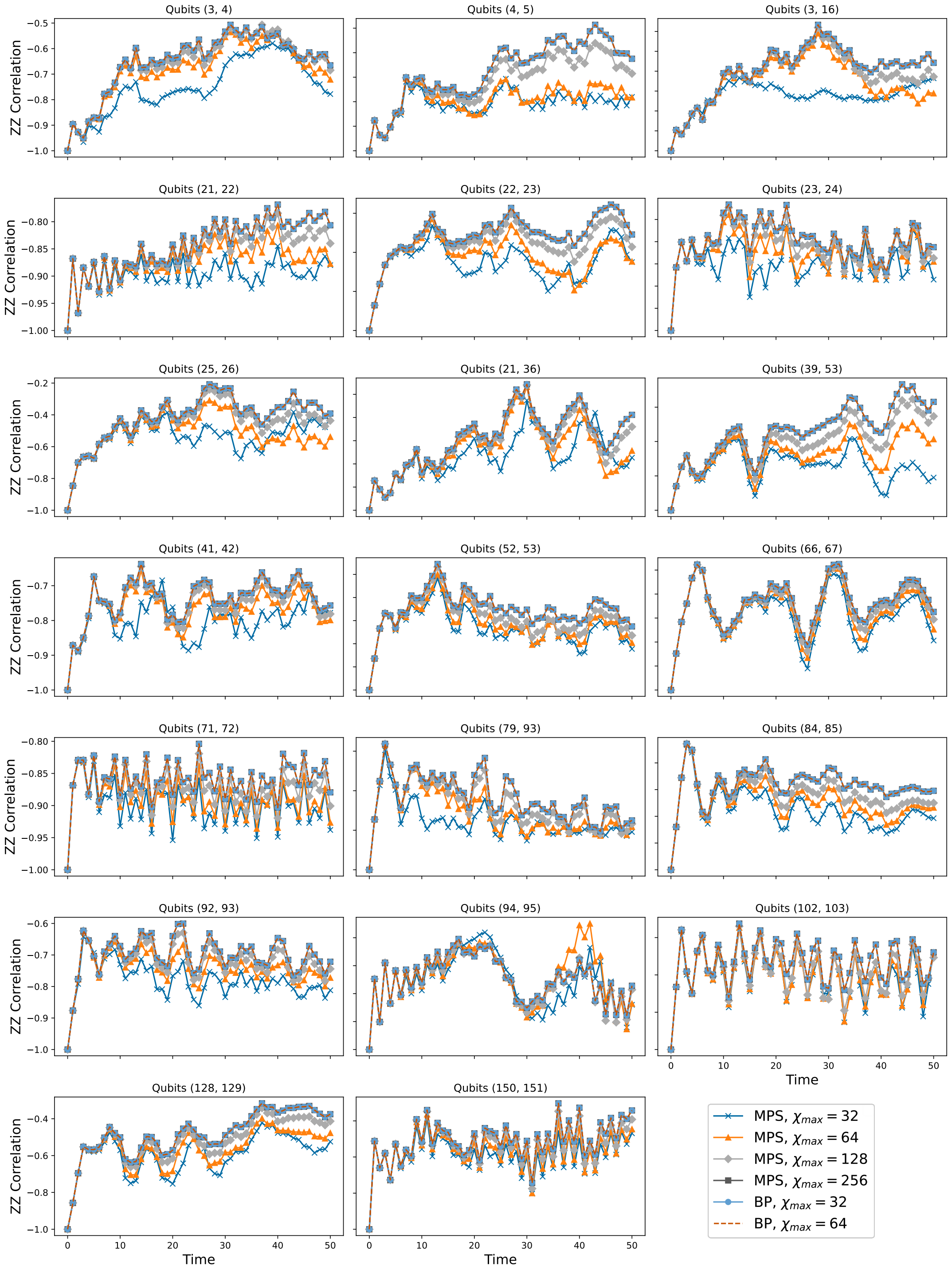

Figure S11. Classical simulation results for ZZ-correlations between a selection of nearest-neighbor sites in the 3x7 geometry for $\epsilon = 0.05$ and $\phi = 0.45\pi$. Similarly to the polarizations in Figure S10, the simulations based on two-dimensional tensor network states with Belief Propagation (BP) converge with increasing bond dimension, whereas the MPS-based simulations don't always converge with the bond dimensions used here, particularly at later times.

and error cancellation[15]. However, even with this full arsenal of techniques, achieving reliable results beyond T_1/T_2 timescales remains difficult. In what follows, we explore an alternative, physics-inspired approach that facilitates the extraction of collective observables — specifically, the average of multiple $s_i(t)$ — for circuit depths up to 450. This

method relies on the following simplified noise model,

$$\tilde{s}_i(t) = f_i(t)s_i(t) + \delta_i(\epsilon, \phi, t), \tag{S.3}$$

which implies that the exact expectation value s_i is related to the experimentally measured $\tilde{s}_i$ through a linear model with an unknown attenuation coefficient f_i and an additive bias term δ_i. Here, $f_i(t)$ accounts for the unbiased, depolarizing component of the noise, while $\delta_i(\epsilon, \phi, t)$ represents the bias arising from relaxation processes. This model effectively approximates sufficiently dense circuits as converting local noise into global white noise.

In what follows, we do not assume a specific parametric form for $f_i(t)$ (e.g., an exponential), but we neglect its dependence on the parameters ϵ and ϕ. In this assumptions,

$$\begin{aligned}\Delta_{\text{noisy}}(\epsilon, \phi, t) &= \frac{1}{N}\sum_{i=1}^{N} s_i(0)\tilde{s}_i(t) = \frac{1}{N}\sum_{k=1}^{N} f_i(t)s_i(0)s_i(t) + \frac{1}{N}\sum_{k=1}^{N} s_i(0)\delta_i(\epsilon, \phi, t) \\ &= \frac{1}{N}\sum_{i=1}^{N}(f_i(t) - f(t))s_i(0)s_i(t) + f(t)\frac{1}{N}\sum_{i=1}^{N} s_i(0)s_i(t) + \frac{1}{N}\sum_{i=1}^{N} s_i(0)\delta_i(\epsilon, \phi, t) \\ &= f(t)\Delta(\epsilon, \phi, t) + \delta(\epsilon, \phi, t),\end{aligned} \tag{S.4}$$

where we have defined,

$$f(t) := \frac{1}{N}\sum_{i=1}^{N} f_i(t), \quad \delta(\epsilon, \phi, t) := \frac{1}{N}\sum_{k=1}^{N} s_i(0)\delta_i(\epsilon, \phi, t) + \eta(t),$$

using the notation $\eta(t) = \frac{1}{N}\sum_{i=1}^{N}(f_i(t) - f(t))s_i(0)s_i(t)$. Next, we assume that the signal attenuation function $f_i(t)$ is statistically independent of the noiseless spin values. This approximation means that we can introduce two random variables, Ξ and Θ, such that $f_1(t) - f(t), \dots, f_N(t) - f(t)$ are observed realizations of Ξ, and $s_1(0)s_1(t), \dots, s_N(0)s_N(t)$ – are realizations of Θ. Since Ξ and Θ are independent, and by the law of large numbers, $\lim_{N\to\infty} \eta(t) = \mathbb{E}(\Xi)\mathbb{E}(\Theta) = 0$ since $\mathbb{E}(\Xi) = 0$. Moreover, using central limit theorem it is not hard to see that $\eta(t) = O\left(\frac{1}{\sqrt{N}}\right)$ for large N and can be neglected. As the result, we get the model,

$$\Delta_{\text{noisy}}(\epsilon, \phi, t) = f(t)\Delta(\epsilon, \phi, t) + \delta(\epsilon, \phi, t), \tag{S.5}$$

where $f(t)$ is the overall decay factor and $\delta(\epsilon, \phi, t)$ is the offset. It originates from the non-equivalence of logical zero and one states that are usually represented by the lowest and first excited level of the superconducting circuit. Since we study the dynamics that breaks the discrete time translation symmetry, we assume that $\delta(\epsilon, \phi, t) = \delta(\epsilon, \phi, t + 2T)$. This is equivalent to the statement that it takes the form,

$$\delta(\epsilon, \phi, t) = \begin{cases} \delta_0(\epsilon, \phi) & \text{if} \quad t \in 2\mathbb{Z} \\ \delta_1(\epsilon, \phi) & \text{if} \quad t \in 2\mathbb{Z} + 1 \end{cases}. \tag{S.6}$$

Then, using the approximations in Eqs. (S.5) for two points (ϵ, ϕ) and $(0, \phi_0)$, we arrive at the expression,

$$\hat{\Delta}(\epsilon, \phi, t) = \Delta(0, \phi_0, t)\frac{\Delta_{\text{noisy}}(\epsilon, \phi, t) - \delta(\epsilon, \phi, t)}{\Delta_{\text{noisy}}(0, \phi_0, t) - \delta(0, \phi_0, t)}. \tag{S.7}$$

It is convenient to choose ϕ_0 corresponding to the closest Clifford point. This means we take $\phi_0 = 0$ for any $\phi \leq \pi/4$ and $\phi_0 = \pi/2$ for all $\phi > \pi/4$.

The expression in Eq. (S.7) can be used to recover the noiseless value, given that the parameters $\delta_{0,1}(0, \phi_0)$ and $\delta_{0,1}(0, \phi_0)$ are known. Unfortunately, these values depend on the noise model and the measurement noise of the device. We may assume, however, that these values weakly depend on the system size as the number of qubits increases due to self averaging. This provides an opportunity to learn these parameters from smaller system sizes, where classical algorithms achieve sufficiently high precision, and then extrapolate to larger two-dimensional systems with 100+ qubits, where these algorithms become unreliable. In what follows we learn offsets $\vec{\delta} = (\delta_0(\epsilon, \phi), \delta_1(\epsilon, \phi), \delta_0(0, \phi_0), \delta_1(0, \phi_0))^\top$ by simulating $\Delta(\epsilon, \phi, t)$ classically, $\Delta_{\text{sim}}(\epsilon, \phi, t)$ and then solving the following convex optimization problem,

$$\min_{\vec{\delta}} J(\vec{\delta}) = \sum_{t=1}^{T}(\Delta_{\text{sim}}(\epsilon, \phi, t) - \hat{\Delta}(\epsilon, \phi, t))^2 + q\|\vec{\delta}\|_2^2, \tag{S.8}$$

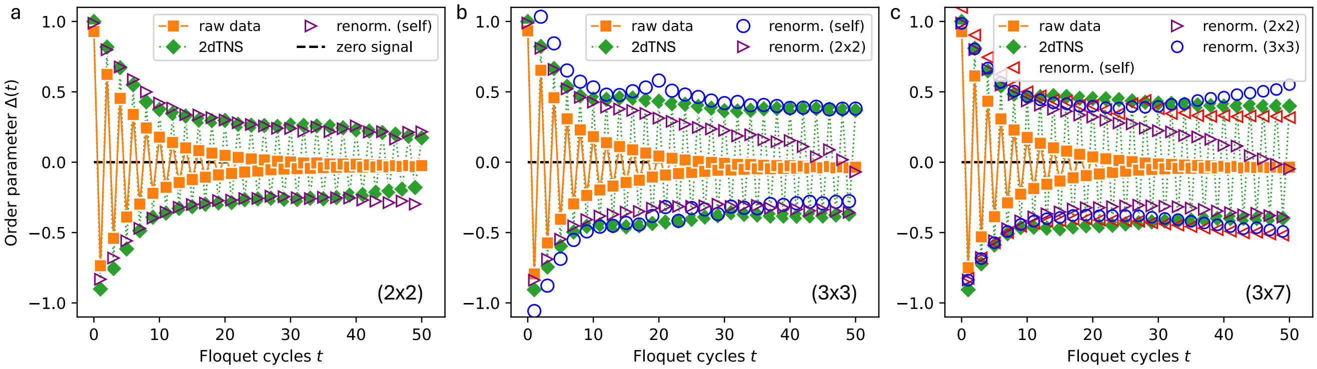

Figure S12. **Renormalization of the order parameter.** Comparison between experimental raw data, numerical simulations (2dTNS with bond dimension $\chi = 128$), and renormalized data. **a**, Time dependence of the $\Delta(t)$ parameter for 2×2 system, measured experimentally using Eq. (S.2) (orange, squares), computed numerically using the exact formula in Eq. (S.1) (green, diamonds), and renormalized using Eq. (S.7) (purple, right triangles). **b**, The same spin ordering parameter for a 3×3 system. The normalized curve shows recovered data using Eq. (S.7) with parameters learned from a 2×2 system (purple, right triangles) and from the system itself (blue, circles). **c**, The same ordering parameters for a 3×7 system. Renormalized curves represent recovered data using Eq. (S.7) with parameters learned from a 2×2 system (purple, right triangles), a 3×3 system (blue, circles), and the system itself (red, left triangles).

where $q > 0$ is a regularization parameter chosen to pick offsets uniquely, with minimal euclidean norm $\| \cdot \|_2^2$.

In the present series of experiments, the results obtained from the optimization of the expression in Eq. (S.8) for a classically simulable, smaller qubit subset (e.g., 2×2) are utilized to mitigate the results for a larger qubit subset (e.g., 3×7), where reliable classical simulation is not feasible. The plots shown in Figs. 2a and 4b in the main text are generated using this approach. More data is shown in Fig. S12. The performance of the proposed method is evaluated using examples of systems of sizes 2×2 (35 qubits) and 3×3 (68 qubits), see Fig. S4 and S5, as well as 3×7 (144 qubits, see Fig. 1b in the main text): the vector of offsets $\vec{\delta}$ is learned by minimizing J with $\Delta_{\rm sim}(\epsilon, \phi, t)$ simulated on 2×2 with parameters $\epsilon = 0.05$ and $\phi = 0.45\pi$. Then this offset is used to compute $\hat{\Delta}(0.05, 0.45, t)$ for 3×3, see Fig. S12 (middle panel), and for 3×7, see Fig. S12 (right panel) for 40 cycles.

However, this method has certain limitations. Specifically, if the learned values of $\delta_k(0, \phi_0, t)$ and $\delta_k(\epsilon, \phi_0, t)$ for the smaller subset differ significantly from their counterparts for the larger subset, this can lead to a scenario where the denominator of Eq. (S.7) is underestimated or even approaches zero. This discrepancy also explains the noticeable deviation in Fig. S12 or signal increase observed at late times Figs. 2a and 4b in the main text.

B. Correlations

A similar approach can be employed to mitigate the spin-spin ordering parameter. The noiseless spin-spin ordering parameter, defined over the set of spin pairs S, is given by

$$\chi = \frac{1}{|S|} \sum_{(i,j) \in S} \langle Z_i Z_j \rangle^2 \equiv \mathbb{E}_S \langle Z_i Z_j \rangle^2, \tag{S.9}$$

where $|S|$ denotes the size of the set S, and $\mathbb{E}_S(\cdot) := \frac{1}{|S|} \sum_{(i,j) \in S}(\cdot)$ represents the expectation value over the set S. In contrast, the noisy counterpart of this parameter can be expressed as

$$\chi_{\rm noisy} := \frac{1}{|S|} \sum_{(i,j) \in S} \langle Z_i Z_j \rangle^2_{\rm noisy} \equiv \mathbb{E}_S \langle Z_i Z_j \rangle^2_{\rm noisy}, \tag{S.10}$$

where $\langle Z_i Z_j \rangle_{\rm noisy}$ denotes the expectation value of the correlator, obtained after noisy evolution and imperfect measurements. Analogous to the treatment of individual spin polarizations, the impact of noise in the dynamics can be represented as

$$\langle Z_i Z_j \rangle_{\rm noisy} = \varphi_{ij}(t) \langle Z_i Z_j \rangle + \eta_{ij}(t), \tag{S.11}$$

where the function $\varphi_{ij}(t)$ characterizes the depolarizing component of the noise, and $\eta_{ij}(t)$ accounts for the noise bias.

The spin glass order parameter is then expressed as,

$$\begin{aligned}\chi_{\text{noisy}}(t) &:= \mathbb{E}_S\langle Z_iZ_j\rangle^2_{\text{noisy}} = \mathbb{E}_S\Big(\varphi_{ij}(t)\langle Z_iZ_j\rangle + \eta_{ij}(t)\Big)^2 \\ &= \Big(\mathbb{E}_S\varphi^2_{ij}(t)\langle Z_iZ_j\rangle^2 + 2\mathbb{E}_S\varphi_{ij}(t)\eta_{ij}(t)\langle Z_iZ_j\rangle + \mathbb{E}_S\eta^2_{ij}(t)\Big).\end{aligned} \tag{S.12}$$

First, assuming that the *noiseless* expectation values are statistically independent of the prefactor $\varphi_{ij}(t)$, we can decouple the correlation function and rewrite the first term in the sum as,

$$\begin{aligned}\mathbb{E}_S\Big[\varphi^2_{ij}(t)\langle Z_iZ_j\rangle^2\Big] &\approx \mathbb{E}_S\big[\varphi^2_{ij}(t)\big]\mathbb{E}_S\big[\langle Z_{i'}Z_{j'}\rangle^2\big] + O\Big(\frac{1}{\sqrt{|S|}}\Big) \\ &= \mathbb{E}_S\big[\varphi^2_{ij}(t)\big]\chi(t) + O\Big(\frac{1}{\sqrt{|S|}}\Big),\end{aligned} \tag{S.13}$$

where we used the definition of the noiseless spin-spin ordering parameter. Similarly, assuming independence between $\varphi_{ij}(t)$ and $\eta_{ij}(t)$, we can rewrite,

$$\begin{aligned}\mathbb{E}_S\Big[\varphi_{ij}(t)\langle Z_iZ_j\rangle\eta_{ij}(t)\Big] &\approx \mathbb{E}_S\big[\varphi_{ij}(t)\langle Z_iZ_j\rangle\big]\mathbb{E}_S\big[\eta_{ij}(t)\big] + O\Big(\frac{1}{\sqrt{|S|}}\Big) \\ &= C_{\text{noisy}}(t)\mathbb{E}_S\big[\eta_{ij}(t)\big] - \Big(\mathbb{E}_S\big[\eta_{ij}(t)\big]\Big)^2 + O\Big(\frac{1}{\sqrt{|S|}}\Big),\end{aligned} \tag{S.14}$$

where we have introduced a new quantity that can be obtained from the experiment,

$$C_{\text{noisy}}(t) := \frac{1}{|S|}\sum_{i\neq j}\langle Z_iZ_j\rangle_{\text{noisy}} \equiv \mathbb{E}_S\langle Z_iZ_j\rangle_{\text{noisy}}. \tag{S.15}$$

Combining the expressions from Eqs. (S.13) and (S.14), we arrive at the approximation for the noisy SG order parameter,

$$\chi_{\text{noisy}}(t) = (N-1)\Big(\mathbb{E}_S\varphi^2_{ij}(t)\chi_{\text{noisy}}(t) + 2\mathbb{E}_S\eta_{ij}(t)C_{\text{noisy}}(t) + \mathbb{E}_S\eta^2_{ij}(t) - \big(\mathbb{E}_S\eta_{ij}(t)\big)^2\Big). \tag{S.16}$$

Restoring the explicit dependence of all parameters on the physical variables ϵ and ϕ, this expression can be written in a more compact form as,

$$\chi_{\text{noisy}}(\epsilon,\phi,t) = \varphi(\epsilon,\phi,t)\chi(\epsilon,\phi,t) - c_1(\epsilon,\phi)C_{\text{noisy}}(\epsilon,\phi,t) - c_2(\epsilon,\phi), \tag{S.17}$$

using the following notations,

$$c_1(\epsilon,\phi) := -2\mathbb{E}_S\eta_{ij}(t)\Big|_{\epsilon,\phi}, \qquad c_2(\epsilon,\phi) := \Big[2\big(\mathbb{E}_S\eta_{ij}(t)\big)^2 - \mathbb{E}_S\eta^2_{ij}(t)\Big]\Big|_{\epsilon,\phi}. \tag{S.18}$$

As a final step, similar to the previous section, we neglect the dependence of the collective parameter $\varphi(\epsilon,\phi,t)$ on the physical parameters ϵ and ϕ, and write it as,

$$\varphi(\epsilon,\phi,t) \approx \varphi(t). \tag{S.19}$$

Using this property and the expression in Eq. (S.17) for parameters $\epsilon = 0$ and $\phi = \phi_0$, we obtain,

$$\chi(\epsilon,\phi,t) = \frac{\chi_{\text{noisy}}(\epsilon,\phi,t) + 2c_1(\epsilon,\phi)C_{\text{noisy}}(\epsilon,\phi,t) + (N-1)c_2(\epsilon,\phi)}{\chi_{\text{noisy}}(0,\phi_0,t) + 2c_1(0,\phi_0)C_{\text{noisy}}(0,\phi_0,t) + (N-1)c_2(0,\phi_0)}, \tag{S.20}$$

where we have taken into account that $\chi(0,\phi_0,t) = 1$.

C. Hamming distance

We recall that the Hamming distance between the input state with polarizations $\mathbf{z} = \{s_i\}$ and the output string $\mathbf{z}' = \{s'_i\}$ is defined as,

$$d(\mathbf{z}, \mathbf{z}') := \frac{1}{2}\sum_{i=1}^{N} |s_i - s'_i| = \frac{1}{2}\sum_{i=1}^{N}(1 - s_i s'_i), \tag{S.21}$$

which shows the number of spin flips required to transform one product state into the other. Correspondingly, the time-dependent noiseless distribution of Hamming distances can be defined,

$$\Phi_d(t) = \mathrm{Prob}\big[d(\mathbf{z}(t), \mathbf{z}(0)) = d\big]. \tag{S.22}$$

The noisy distribution, obtained for the noisy output $z_{\mathrm{noisy}}(t) = \langle Z_i(t)\rangle_{\mathrm{noisy}}$, can be derived from the original noiseless distribution by applying a certain transformation represented by a time-dependent kernel $K(d, d'|t)$,

$$\Phi_d^{\mathrm{noisy}}(t) := \mathrm{Prob}\big[d(\mathbf{z}_{\mathrm{noisy}}(t), \mathbf{z}(0)) = d\big] = \sum_{d'=0}^{N} K(d, d'|t)\Phi_{d'}(t). \tag{S.23}$$

While the exact form of this kernel is unknown and hard to derive, one could make an empirical approximation to it. One such approximation is to consider this kernel as a kernel $T_p(d, d')$ of a transformation that flips each spin independently with a certain time-dependent probability p, i.e.,

$$K(d, d'|t) \approx T_p(d, d')\Big|_{p=p(t)}. \tag{S.24}$$

A straightforward derivation provides an expression for this kernel in the form,

$$T_p(d, d') = \sum_{\max(0, d+d'-N)}^{\min(d,d')} C_x^{d'} C_{d-x}^{n-d'} p^{d+d'-2x}(1-p)^{N+2x-d-d'}. \tag{S.25}$$

The parameter p is unknown. It can be derived for a relevant experimental point if we neglect the dependence of $p(t)$ on the parameters ϵ and ϕ. Then, this parameter can be learned from observing the Clifford point. This learning procedure is formulated as an optimization task,

$$p(t) = \mathrm{argmin}[L_1(p, t)], \qquad L_1(p, t) = \sum_{d=0}^{N}\Big(\Phi_d^{\mathrm{noisy}}(t)\Big|_{\epsilon=0, \phi=\phi_0} - T_p\big(d, d_{\mathrm{cliff}}(t)\big)\Big)^2. \tag{S.26}$$

The dependence of the learned value of $p(t)$ as a function of the depth t is shown in Fig. S13a. The comparison between the experimental distribution and the distribution obtained using the kernel $T_p(d, d')$ is shown in Fig. S13b.

With the knowledge of the kernel $T_p(d, d')$, it is possible to "reverse" the effect of noise and learn the noiseless distribution. However, since the kernel has many eigenvalues that are close to zero, the outcome will depend significantly on small perturbations of the noisy distribution. To avoid this problem, we restrict the space of possible outputs by considering a fixed form of the noiseless distribution. In particular, we look only at distributions that take the form,

$$\Phi_d^{\mathrm{trial}} = A\frac{\exp\left(-\frac{(d-d_0)^2}{2\sigma^2}\right)}{1 + \exp(kd + q)} \tag{S.27}$$

where d_0, σ, k, and q are free parameters, and A is the normalization factor that depends on them.

We determine the most suitable trial distribution by minimizing the loss function,

$$\Phi_d^{\mathrm{trial}} = \mathrm{argmin}[L_2], \qquad L_2 = \sum_{d=0}^{N}\Big(\Phi_d^{\mathrm{noisy}}(t) - \sum_{d'=0}^{N} T_p(d, d')\Phi_{d'}^{\mathrm{trial}}\Big)^2 + \lambda_1(\mu_{\mathrm{trial}} - \mu(t))^2 + \lambda_2(\sigma_{\mathrm{trial}} - \sigma(t))^2, \tag{S.28}$$

where λ_1 and λ_2 are positive parameters, μ_{trial} and σ_{trial} are the mean value and variance of the distribution Φ_d^{trial}, while $\mu(t)$ and $\sigma(t)$ are the best estimates for the mean value and variance of the true noiseless distribution. The mean value is connected to the order parameter as,

$$\mu(t) := \Big\langle \frac{1}{2}\sum_i \Big(1 - s_i(0)Z_i(t)\Big)\Big\rangle = \frac{N}{2}\Big(1 - \Delta(t)\Big), \tag{S.29}$$

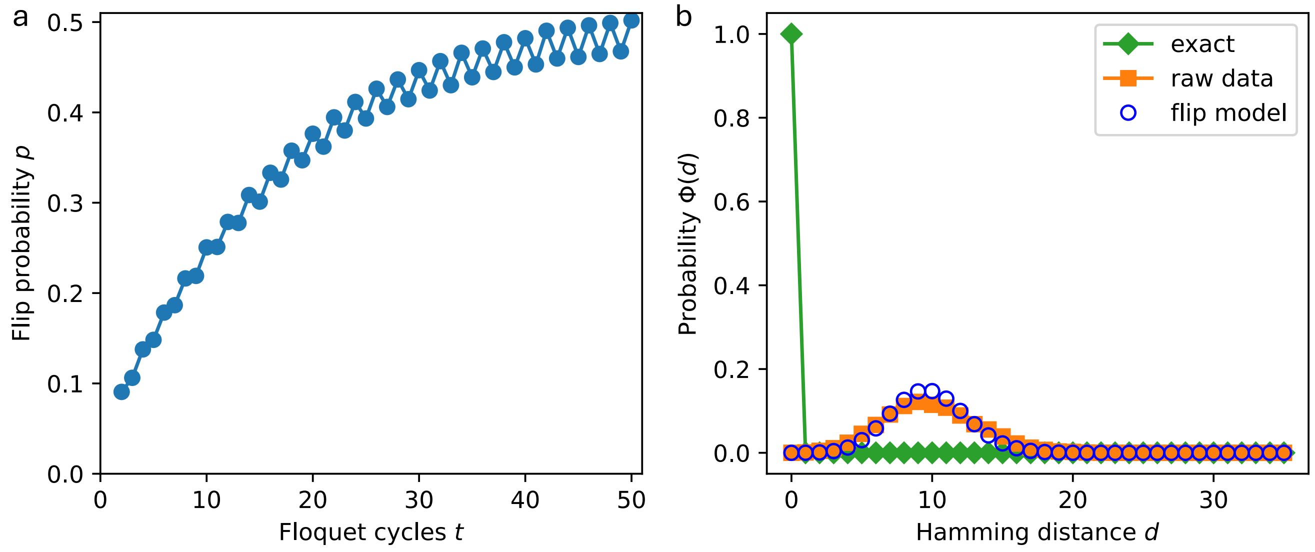

Figure S13. **Noise filtering for Hamming distance.** **a**, The learned flip parameter $p(t)$ as a function of the depth t for the 2×2 system, obtained through the optimization procedure in Eq. (S.26). **b**, Comparison of the experimental noisy distribution (orange, squares) with the distribution obtained by applying the kernel T_p (blue, circles) to the true distribution (green, diamonds).

where $\Delta(t)$ is the order parameter defined above in Eq. (S.1). The variance, in turn, can be expressed as,

$$\sigma(t) := \frac{1}{4}\Big\langle\Big(\sum_i s_i(0)\big[Z_i(t) - \langle Z_i(t)\rangle\big]\Big)^2\Big\rangle = \frac{1}{4}\sum_{ij} s_i(0)z_j(0)\Big(\langle Z_i(t)Z_j(t)\rangle - \langle Z_i(t)\rangle\langle Z_j(t)\rangle\Big). \tag{S.30}$$

This expression represents the quantum Fisher information.

IV. TRENDS IN ORDER PARAMETERS DERIVED FROM RAW DATA

A. Quantum Fisher information order parameter

In our analysis of the Hamming distributions of Section III C, we define the variance of the Hamming distance distribution in Eq. (S.30), which corresponds with the quantum Fisher information order parameter $F_Q(t)$. Separating out the contributions of the diagonal elements, we obtain

$$F_Q(t) = 1 - \frac{1}{N}\sum_{i=1}^{N} \langle Z_i\rangle_t^2 + \frac{1}{N}\sum_{i\neq j} C_{ij}(t). \tag{S.31}$$

where $C_{ij}(t) = \langle Z_i Z_j\rangle_t - \langle Z_i\rangle_t \langle Z_j\rangle_t$ is two-point correlation function. The first term corresponds to $F_Q = 1$, indicating the vanishing of local observables, e.g., due to thermalization or noise. The second term is always negative and corresponds to persistent independent spin polarizations in the Z direction, while the third term is always positive and represents the spread of correlations in the system. At early times, the system is polarized and uncorrelated, which leads to $F_Q = 0$. As the system evolves, spins depolarize, making the contribution of the second term smaller, while simultaneously correlations develop, leading to $F_Q > 0$ at $t > 0$. In the ergodic phase, one can expect that both polarization and correlations vanish rapidly, resulting in $F_Q \to 1$ after a few cycles. In contrast, in a DTC regime one may expect that the correlations remain sufficiently strong and the sum of the polarization and correlation terms remains positive (assisted by the relative number of terms between the two contributions: N terms in the polarization sum and $N(N-1)$ terms in the correlator sum). The values of $F_Q > 1$ at later times shown in Fig. 3c in the main text appear to be the result of persistent system-wide correlations, characteristic of the DTC regime.

For parameter ranges where the MBL and DTC regimes, identified by the two-time correlator, are shared by both the Néel (Fig. 3 in the main text) and polarized (Fig. 4 in the main text) initial states, noisy $F_Q(t)$ exhibits

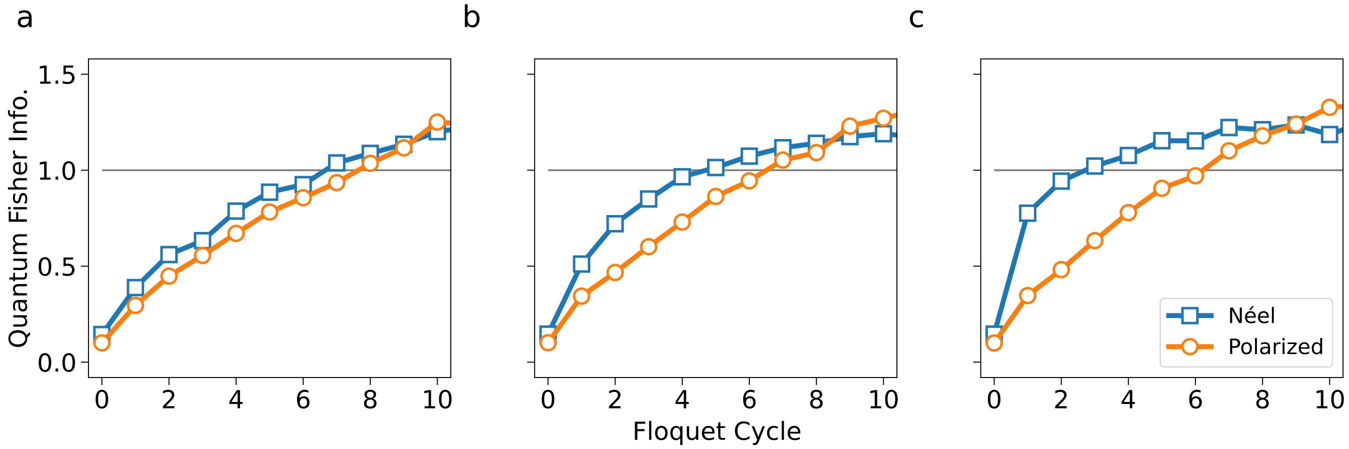

Figure S14. $F_Q(t)$, given in Eq. (S.30), derived from experimental raw data for the Néel and polarized initial states, at the (ϕ, ϵ) points on the phase diagram located at **a,** $(0.0, 0.0)$, **b,** $(0.0, 0.05)$, and **c,** $(0.0, 0.1)$. The shot noise limit at $F_Q = 1$ (gray line), is added for clarity.

logarithmic growth. This behavior is remarkable given that these parameter ranges encompass unperturbed ($\epsilon = 0$) and perturbed ($\epsilon > 0$) kicked Ising models. The curvature of $F_Q(t)$ displays a pronounced dependence on the initial state when spin-flip components are introduced ($\epsilon > 0$), as shown in the experimental data of Fig. S14. For Floquet cycles prior to complete depolarization, the kicked Ising model in Fig. S14a shows little discernible difference between the Néel and polarized initial states as in both cases its growth originates from the underlying noise. In contrast, at values of ϵ that perturbatively move the system into the Heisenberg-perturbed regime, Fig. S14b,c, the curvature of F_Q differs dramatically between these two initial states.

B. Time correlation order parameter

The presence of subharmonic responses in local observables under a periodic drive is one of the primary indicators of discrete time-crystalline behavior. A Fourier transform of these observables, such as the two-time correlator order parameter defined in Eq. (5) in the main text, reveals these subharmonic responses through a pronounced peak at characteristic frequency $\omega = \omega_D/2$, where ω_D is the drive frequency. In the main text, evidence of such a subharmonic response is observed across a variety of parameter regimes and initial states, including both the Néel (Fig. 3) and polarized (Fig. 4) initial states. Notably, the polarized initial state exhibits a robust subharmonic signal over a wider range of spin-flip couplings ϵ compared to the Néel initial state. Other initial states do not display this stability and behave nearly similarly to the Néel initial state. In Fig. S15, the Fourier transforms of the two-time correlator order parameter, derived from experimental data, are shown for several random initial states alongside those for the Néel and polarized initial states. Each random state is generated as a product state of independently and randomly assigned maximally polarized spins $s_i \in \{-1, 1\}$. Near the Clifford point in Fig. S15a, the peak at $\omega = \omega_D/2$ is pronounced for all initial states, though the polarized initial state shows a larger amplitude than the other states. As the parameters are tuned away from the integrable point, by decreasing the kick-angle ϕ and increasing spin-flip strength ϵ, the polarized state retains a half-frequency peak, in contrast to the other initial states, see Fig. S15b,c. In Fig. S15c, as the kick angle approaches the ergodic regime at $\phi = \pi/4$ the original half-frequency peaks disappears, indicating the cessation of time-crystalline behavior.

C. Edwards-Anderson spin glass order parameter

As described in the main text, if the set of spin pairs M in Eq. (6) is expanded to all qubit pairs, one recovers an Edwards-Anderson spin glass order parameter

$$\chi_{\mathrm{SG}}(t) = \frac{1}{N(N-1)} \sum_{i,j} \langle Z_i Z_j \rangle_t^2 \,. \tag{S.32}$$

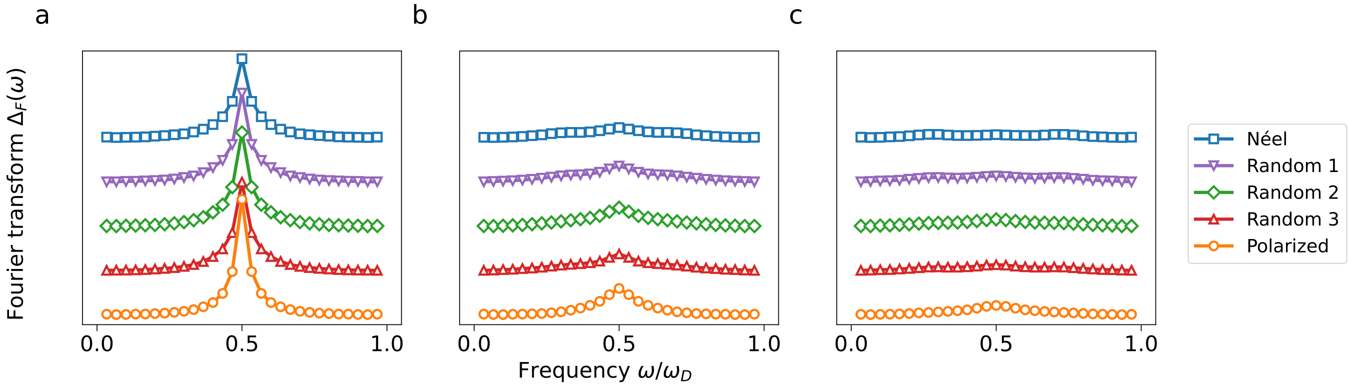

Figure S15. The Fourier transform of the order parameter $\Delta(t)$, defined in Eq. (5) in the main text and derived from experimental raw data is shown as a function of the frequency ω in units of the drive frequency ω_D. The components for $\omega > \omega_D/2$ represent the original spectrum folded into this band. Different initial states are plotted, which include the Néel (blue squares) and polarized (orange circles) initial states, and three realizations of random initial states (purple inverted triangles, green diamonds, and red triangles), as described in Section IV B. Panels correspond to points on the phase diagram (ϕ, ϵ) located at **a,** $(0.45\pi, 0.05)$, **b,** $(0.35\pi, 0.15)$, and **c,** $(0.3\pi, 0.2)$. Curves for different initial states exhibit a vertical displacement for illustration purposes.

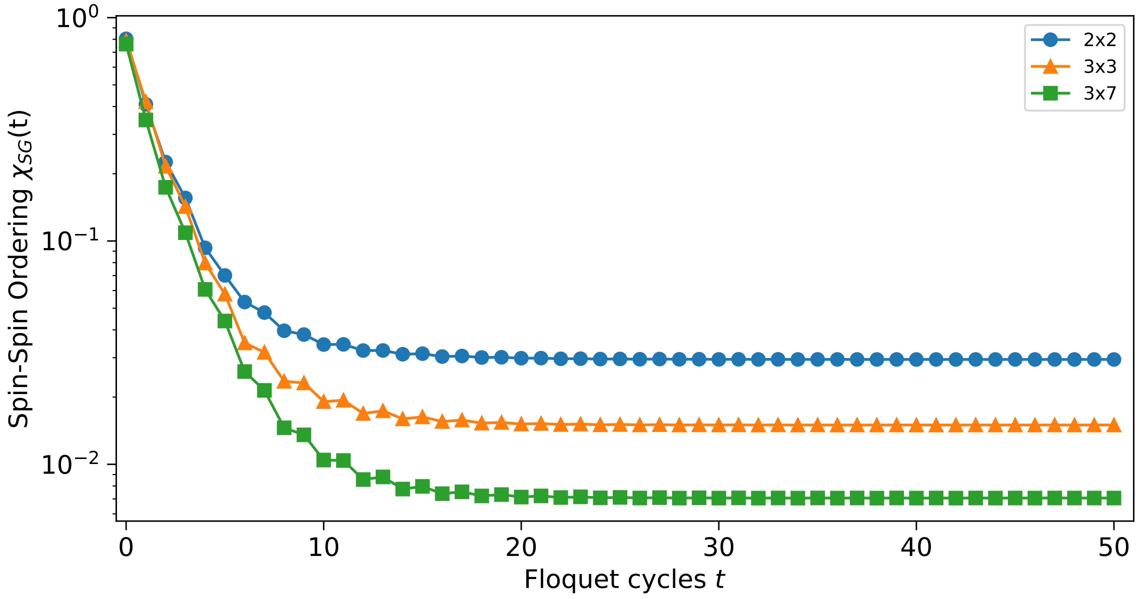

Figure S16. Comparison of the Edwards-Anderson spin glass order parameter, Eq. (S.32), derived from experimental raw data for the 2×2, 3×3, and 3×7 geometries at $\phi = 0.45\pi$, $\epsilon = 0.05$.

The raw values of χ_{SG} are shown in Fig. S16, and are similar to the raw data shown in Fig. 2b in the main text. The parameter decays due to vanishing correlators $\langle Z_i Z_j \rangle |_{i \neq j}$ until it reaches a trivial $1/N$ value coming from the diagonal terms $\langle Z_i Z_i \rangle \equiv \langle I \rangle = 1$ in Eq. (S.32). We note that because of the noisy data, a zero value of this parameter from raw experimental data (even for the large N investigated in this work) is not a sufficient condition to see the actual phase of the system.

V. DISCRETE VS CONTINUOUS TIME

The Floquet unitary - defined in Eq. (1) in the main text - which governs the dynamics of each cycle is made up of two parts: the Heisenberg-like evolution $U_F = U^{(3)}_{XXZ} U^{(2)}_{XXZ} U^{(1)}_{XXZ}$ and the kick-pulse $U_{\rm X} = \prod_{i=1}^{N} \exp(-i\phi X_i)$. The

operator U_F can be viewed as a Trotterization of the continuous-time evolution operator:

$$U_{XXZ} = \exp\left(-iT\sum_{(i,j)\in G} J_{ij}(\epsilon X_iX_j + \epsilon Y_iY_j + Z_iZ_j)\right) \tag{S.33}$$

More precisely, we can define a general Trotterized version of the continuous-time evolution operator U_{XXZ} with k steps per cycle:

$$\begin{aligned} U_{XXZ,\ k} = [&\prod_{(i,j)\in G_1} \exp\left(-i\frac{T}{k}J_{ij}(\epsilon X_iX_j + \epsilon Y_iY_j + Z_iZ_j)\right) \\ &\prod_{(i,j)\in G_2} \exp\left(-i\frac{T}{k}J_{ij}(\epsilon X_iX_j + \epsilon Y_iY_j + Z_iZ_j)\right) \\ &\prod_{(i,j)\in G_3} \exp\left(-i\frac{T}{k}J_{ij}(\epsilon X_iX_j + \epsilon Y_iY_j + Z_iZ_j)\right)]^k \end{aligned} \tag{S.34}$$

such that $U_{XXZ,\ k} \to U_{XXZ}$ as $k \to \infty$. The model in Eq. (1) in the main text is a special case of (S.34) with $T = 1$ and $k = 1$ steps per cycle. In Figure S17, we consider the effect of different values of k on the order parameter Δ_{MBL} for a 10-cycle simulation on 3×3 heavy hexagons (68 qubits). We plot the raw data, the renormalized data and the classical data obtained from the 2D TNS simulations. We can see that for all of these curves, the results have not yet converged in k, suggesting that one may need to consider values of k higher than 4 to understand the full phase diagram of the model with continuous time. To do so on a digital quantum device, one may use Multiproduct Formulas[16–18] - see upcoming work.

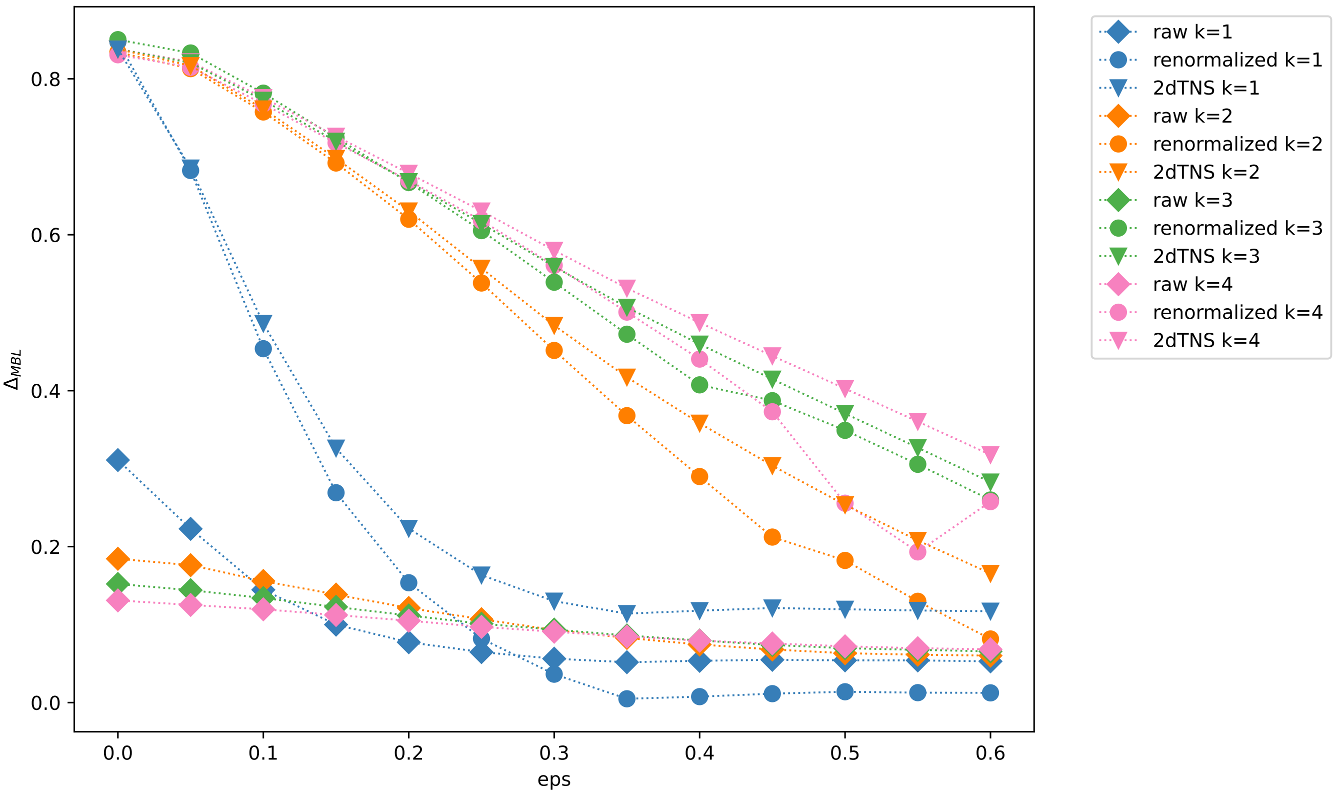

Figure S17. **Comparing the effect of different Trotter step sizes.** We plot the order parameter Δ_{MBL} vs ϵ with the X-gate angle is fixed to $\phi = 0.45\pi$. Results in this plot correspond to 3×3 heavy hexagons (68 qubits) and 10 cycles. The curves have not yet converged at $k = 4$ Trotter steps per cycle for this part of the phase diagram.

Supplementary References

1. Tindall, J. & Fishman, M. Gauging tensor networks with belief propagation. *SciPost Phys.* **15**, 222 (2023).
2. Tindall, J., Fishman, M., Stoudenmire, E. M. & Sels, D. Efficient tensor network simulation of ibm's eagle kicked ising experiment. *PRX Quantum* **5**, 010308 (2024).
3. Schollwöck, U. The density-matrix renormalization group in the age of matrix product states. *Ann. of Phys.* **326**, 96–192 (2011).
4. Vidal, G. Efficient simulation of one-dimensional quantum many-body systems. *Phys. Rev. Lett.* **93**, 040502 (2004).
5. Haegeman, J. *et al.* Time-dependent variational principle for quantum lattices. *Phys. Rev. Lett.* **107**, 070601 (2011).
6. Haegeman, J., Lubich, C., Oseledets, I., Vandereycken, B. & Verstraete, F. Unifying time evolution and optimization with matrix product states. *Phys. Rev. B* **94**, 165116 (2016).
7. Zaletel, M. P., Mong, R. S. K., Karrasch, C., Moore, J. E. & Pollmann, F. Time-evolving a matrix product state with long-ranged interactions. *Phys. Rev. B* **91**, 165112 (2015).
8. Schuch, N., Wolf, M. M., Verstraete, F. & Cirac, J. I. Computational complexity of projected entangled pair states. *Phys. Rev. Lett.* **98**, 140506 (2007).
9. Evenbly, G. Gauge fixing, canonical forms, and optimal truncations in tensor networks with closed loops. *Phys. Rev. B* **98**, 085155 (2018).
10. Haghshenas, R., O'Rourke, M. J. & Chan, G. K.-L. Conversion of projected entangled pair states into a canonical form. *Phys. Rev. B* **100**, 054404 (2019).
11. Hauschild, J. & Pollmann, F. Efficient numerical simulations with Tensor Networks: Tensor Network Python (TeNPy). *SciPost Phys. Lect. Notes* 5 (2018).
12. Wallman, J. J. & Emerson, J. Noise tailoring for scalable quantum computation via randomized compiling. *Phys. Rev. A* **94**, 052325 (2016).
13. Temme, K., Bravyi, S. & Gambetta, J. M. Error mitigation for short-depth quantum circuits. *Phys. Rev. Lett.* **119**, 180509 (2017).
14. Li, Y. & Benjamin, S. C. Efficient variational quantum simulator incorporating active error minimization. *Phys. Rev. X* **7**, 021050 (2017).
15. Van Den Berg, E., Minev, Z. K., Kandala, A. & Temme, K. Probabilistic error cancellation with sparse pauli–lindblad models on noisy quantum processors. *Nat. Phys.* **19**, 1116–1121 (2023).
16. Zhuk, S., Robertson, N. F. & Bravyi, S. Trotter error bounds and dynamic multi-product formulas for hamiltonian simulation. *Phys. Rev. Research* **6**, 033309 (2024).
17. Vazquez, A. C., Egger, D. J., Ochsner, D. & Woerner, S. Well-conditioned multi-product formulas for hardware-friendly hamiltonian simulation. *Quantum* **7**, 1067 (2023).
18. Robertson, N. F. *et al.* Tensor Network Enhanced Dynamic Multiproduct Formulas. *PRX Quantum* **6**, 020360 (2025).
